# Racial and Ethnic Disparities in Use of Colorectal Cancer Screening Among Adults With Chronic Medical Conditions: BRFSS 2012–2020

**DOI:** 10.5888/pcd21.230257

**Published:** 2024-02-22

**Authors:** Maira A. Castañeda-Avila, Mayra Tisminetzky, Atinuke G. Oyinbo, Kate Lapane

**Affiliations:** 1 Division of Epidemiology, Department of Population and Quantitative Health Sciences, University of Massachusetts Chan Medical School, Worcester, Massachusetts; 2 Division of Health Systems Science, Department of Medicine, University of Massachusetts Chan Medical School, Worcester, Massachusetts

## Abstract

**Introduction:**

People with chronic conditions and people with colorectal cancer (CRC) may share common risk factors; thus, CRC screening is important for people with chronic conditions. We examined racial and ethnic differences in the use of CRC screening among people with various numbers of chronic conditions.

**Methods:**

We included data on adult respondents aged 50 to 75 years from the Behavioral Risk Factor Surveillance System in 2012 through 2020. We categorized counts of 9 conditions as 0, 1, 2, 3, and ≥4. We classified self-reported CRC screening status as up to date or not. We used Poisson models to estimate adjusted prevalence ratios (APRs) among the different counts of chronic conditions in 4 racial and ethnic groups: Hispanic adults with limited English proficiency (LEP), Hispanic adults without LEP, non-Hispanic Black adults, and non-Hispanic White adults.

**Results:**

Overall, 66.5% of respondents were up to date with CRC screening. The prevalence of being up to date increased with the number of chronic conditions. We found disparities among racial and ethnic groups. Hispanic respondents with LEP had lower rates than non-Hispanic White adults of being up to date with CRC screening across all counts of chronic conditions (APR for 0 conditions = 0.67; 95% CI, 0.64–0.71; APR for ≥4 conditions = 0.85; 95% CI, 0.79–0.91). Hispanic respondents without LEP with 0, 1, or 2 conditions were less likely than non-Hispanic White respondents to be up to date with CRC screening. We found no significant differences between non-Hispanic Black and non-Hispanic White respondents.

**Conclusion:**

We found disparities among Hispanic BRFSS respondents with LEP, who had lower rates than non-Hispanic White respondents of being up to date with CRC screening, regardless of the number of chronic conditions. Tailored interventions are needed to address these disparities and improve screening rates, particularly among Hispanic people.

**Summary**

**What is already known on this topic?**

People with chronic medical conditions and people with colorectal cancer (CRC) may share common risk factors, and disparities exist in CRC screening rates, particularly among Hispanic people with limited English proficiency.

**What is added by this report?**

We found disparities in rates of CRC screening among adults with chronic medical conditions: Hispanic adults with limited English proficiency had lower rates than non-Hispanic White adults, regardless of the number of chronic medical conditions.

**What are the implications for public health practice?**

Tailored interventions are warranted to improve CRC screening rates, especially among Hispanic people with language barriers. Addressing health care access, language barriers, and cultural sensitivity are essential to reduce disparities and promote timely CRC screening.

## Introduction

Colorectal cancer (CRC) is the third most diagnosed cancer in the US (1). CRC screening guidelines are strongly supported by effectiveness data, but use of screening in the US is suboptimal (2–5). In 2022, the percentage of Hispanic adults who reported being up to date with CRC screening was lower (61.7%) than among non-Hispanic White (74.6%) and non-Hispanic Black (75.3%) adults (6). In addition, being male, having a low level of education, living in poverty, lacking health insurance, and using English as a second language have been associated with lower rates of CRC screening (7–10).

Adults with chronic medical conditions and adults with CRC may share risk factors such as obesity, physical inactivity, and smoking (11–14). Identifying CRC at an early stage through screening is crucial not only to improve CRC outcomes but also to manage other chronic conditions, which could ultimately improve relevant clinical outcomes (15). Previous studies examined the relationship between the presence of chronic conditions (eg, diabetes, cardiovascular disease, obesity) and CRC screening, but results were inconsistent (16–20). Some studies showed that chronic conditions were associated with lower rates of screening, while others found higher rates (16–19).

It remains uncertain how chronic conditions are associated with CRC screening across racial and ethnic groups. Language barriers pose an obstacle to CRC screening in racial and ethnic minority populations because limited English proficiency (LEP) hinders comprehension of screening guidelines and contributes to misconceptions and insufficient knowledge (8,21,22). We examined the effect of the presence and number of chronic conditions on the use of CRC screening among 4 racial and ethnic groups responding to the BRFSS during 2012 through 2020: non-Hispanic Black, Hispanic (with and without LEP), and non-Hispanic White.

## Methods

We used cross-sectional data from the 2012–2020 BRFSS, which comprises 5 years of survey information in biannual increments (2012, 2014, 2016, 2018, 2020) (23–27). We selected these years because all jurisdictions included the CRC screening module, ensuring that nationally representative inferences could be drawn. The BRFSS database is maintained by the Centers for Disease Control and Prevention and gathers information on a broad spectrum of health-related behaviors and risk factors among US adults, including information on health status, behaviors such as smoking and physical activity, access to health care, demographic characteristics, mental health, and social determinants of health.

BRFSS recruits participants for telephone surveys via random digit dialing of civilian, noninstitutionalized resident adults aged 18 or older in the US by using a multistage household sampling design. Interviews are conducted in all 50 states, plus the District of Columbia and the territories of Guam, Puerto Rico, and the US Virgin Islands. The median (range) survey response rate for all states, the federal district, and all participatory territories was 45.2% (27.7%–60.4%) in 2012, 47.0% (25.1%–60.1%) in 2014, 47.0% (30.7%–65.0%) in 2016, 49.9% (38.8%–67.2%) in 2018, and 47.9% (34.5%–67.2%) in 2020 (23–27).

In 2021, the US Preventive Services Task Force (USPSTF) began recommending CRC screening for adults aged 45 to 49 years (28). However, during the time frame of our study, USPSTF recommended CRC screening for adults aged 50 to 75 years (29). Therefore, we included participants if they were aged 50 to 75 years and identified as non-Hispanic White, non-Hispanic Black, or Hispanic. We excluded participants who reported receiving a previous diagnosis of CRC, had missing data on chronic conditions or CRC screening, or had missing responses for the variables included in the models. The final study population consisted of 989,830 men and women aged 50 to 75 years (weighted n = 80,673,621).

### Operational definition of CRC screening

Our study measured adherence to CRC screening recommendations according to USPSTF guidelines (28). BRFSS survey respondents were asked about their history of fecal occult blood test (FOBT), sigmoidoscopy, and/or colonoscopy, and time since their most recent screening. On the basis of these responses, we developed a composite CRC screening variable that classified participants as either up to date or not up to date with screening recommendations. Participants were classified as up to date if they had performed a home FOBT kit within the previous year, underwent sigmoidoscopy within the previous 5 years, and/or had colonoscopy within the previous 10 years. Participants were considered not up to date if they had received any of the 3 tests outside the recommended time frame or never received any of these tests. Because adults are better able to recall whether they had undergone a preventive test within a given time frame rather than providing the specific dates of screening (30), national surveys such as BRFSS use this approach to determine CRC screening.

### Operational definition of race, ethnicity, and limited English proficiency

The BRFSS survey included 3 questions related to race and ethnicity: 1) “Are you of Hispanic, Latino/a, or Spanish origin?” 2) “Which one or more of the following would you say is your race?” and 3) “Which one of these groups would you say best represents your race?” We used either the reported race and ethnicity or an imputed race and ethnicity. If a respondent refused to indicate a race and ethnicity, BRFSS input the most common race and ethnicity response for that region of the state (31). To explore the role of English language proficiency among Hispanic respondents, we categorized Hispanic adults who responded to the survey in Spanish as having LEP. The number of respondents from other racial and ethnic groups who responded in Spanish or other language was small: 0.8% of non-Hispanic White adults and 0.7% of non-Hispanic Black adults responded in Spanish or another language. Because of these small sample sizes, we did not assess LEP for these groups. Non-Hispanic groups were assumed to have English proficiency. We included the following racial and ethnic groups in the analysis: non-Hispanic White, non-Hispanic Black, and Hispanic with and without LEP.

### Operational definition of chronic medical conditions

We calculated the number of chronic conditions by tallying self-reported data on 9 chronic conditions reported in BRFSS: arthritis (including rheumatoid arthritis, gout, lupus, and fibromyalgia), asthma, cancer, cardiovascular diseases (comprising heart attack, coronary heart disease, and stroke), chronic obstructive pulmonary disease (including emphysema and chronic bronchitis), depression (including depression of any severity, major depression, dysthymia, or minor depression), diabetes (excluding gestational diabetes), kidney disease (excluding kidney stones, bladder infection, and incontinence), and nonmelanoma skin cancer. On the basis of distribution of the counts of chronic conditions, we categorized these conditions as 0, 1, 2, 3, or ≥4.

### Covariates

We included variables that may affect the relationship between race and ethnicity and chronic conditions or CRC screening as covariates. Sociodemographic characteristics were age (<65 and ≥65 y), sex (male and female), educational attainment (less than a high school diploma, high school diploma, and some college or more), marital status (married/living together, divorced or separated, and widowed or single), and employment status (employed/self-employed, homemaker/student, unemployed/unable to work, and retired). We considered the following self-reported clinical characteristics: general health (excellent/very good/good or fair/poor), smoking status (current, former, or never smoker), and binge drinking (men having ≥5 drinks or women having ≥4 drinks on 1 occasion in the past month). We also considered the following health care characteristics: having health insurance coverage (any coverage or no coverage), having a primary care provider or any health care provider (yes or no), reporting financial barriers to health care access (yes or no), and having had a routine checkup within the past 12 months (yes or no).

### Statistical analysis

We performed all statistical analyses in Stata version 16 (StataCorp LLC). We used BRFSS weights to make our results representative of the US adult population aged 50 to 75 years, adjusting for unequal probability of being selected, noncoverage, and nonresponse. We divided weights for data from all years by the number of years of survey data available from each jurisdiction. We used year-specific stratum and primary sample unit (PSU) identifiers in all analyses to avoid treating unrelated observations as coming from related strata or PSUs simply because they were interviewed in different years. We compared sociodemographic, clinical, and health care characteristics between participants with up-to-date and not up-to-date CRC screening status.

To calculate the prevalence ratios (PRs) of CRC screening among Hispanic respondents with and without LEP and non-Hispanic Black respondents versus non-Hispanic White respondents across counts of chronic conditions, we used weighted Poisson regression models. In each model, we added an interaction term between the number of chronic conditions and racial and ethnic group to estimate PRs that compare CRC screening among Hispanic people without LEP and non-Hispanic White respondents, Hispanic respondents with LEP and non-Hispanic White respondents, and non-Hispanic Black respondents and non-Hispanic White respondents at each count of chronic conditions. We used this approach instead of a regular stratified analysis by each racial and ethnic group to make direct comparisons between Hispanic people with and without LEP and non-Hispanic Black respondents who had different numbers of chronic conditions versus non-Hispanic White respondents who had the same number of chronic conditions while adjusting for confounders. We used empirical SEs for valid statistical inferences. We used unadjusted Poisson models to estimate the overall prevalence of CRC screening and adjusted Poisson models to adjust for confounders such as age, sex, health insurance, and survey year. The addition of other potential confounders did not substantially change estimates. We estimated crude and adjusted PRs and 95% CIs from these models. To determine differences between respondents with chronic conditions and respondents without chronic conditions and evaluate the equality of coefficients, we used a weighted, adjusted Wald χ^2^ test.

## Results

The weighted sample included approximately 80 million US adults aged 50 to 75 years. Of the total population, 68.1% were aged 50 to 65 years, 52.3% were women, and 76.4% were non-Hispanic White. Overall, about two-thirds of respondents (66.5%) were up to date with CRC screening guidelines. Among respondents who were not up to date with CRC screening, 78.5% were aged 50 to 65 years. Almost one-fifth (19.2%) who were not up to date had less than a high school diploma, 25.1% reported fair or poor health, and 22.7% were current smokers. Furthermore, 16.5% of those not up to date lacked health insurance coverage, 22.2% had no primary care provider, one-third (33.8%) lacked routine care in the previous year, and 16.4% reported financial barriers to care (Table). Colonoscopy was the most used screening method (63.2%), followed by FOBT (10.2%) and sigmoidoscopy (3.2%). Factors associated with the use of colonoscopy within the previous 10 years were similar to the factors associated with being up to date with any CRC screening.

**Table:** Sociodemographic, Clinical, Health Care, and Behavioral Characteristics, by Use of Colorectal Cancer Screening, Among US Adults Aged 50 to 75 Years, Behavioral Risk Factor Surveillance System, 2012–2020^a^

Characteristic	All	Any colorectal cancer screening	
		Never or not up to date	Up to date
Unweighted no.	989,700	303,382	686,318
Weighted no.	80,673,621	27,015,012	53,658,610
Weighted row %	100	33.5	66.5
Female	52.3	50.0	53.4
Aged ≥65 years	31.9	21.5	37.1
Race and ethnicity			
Hispanic, no limited English proficiency	6.0	7.1	5.5
Hispanic, limited English proficiency^b^	6.1	9.9	4.1
Non-Hispanic Black	11.5	11.4	11.5
Non-Hispanic White	76.4	71.6	78.8
Marital status			
Married/living together	64.7	58.6	67.8
Divorced/separated	18.8	22.4	16.9
Widowed/never married	16.5	19.0	15.3
Education			
Less than high school	13.2	19.2	10.1
High school diploma	28.2	31.1	26.8
Some college or more	58.6	49.7	63.1
Employment status			
Employed	48.8	54.7	45.8
Homemaker/student	5.1	6.3	4.5
Unemployed/unable to work	16.1	19.3	14.4
Retired	30.0	19.7	35.2
Annual household income <$35,000	35.3	44.5	30.7
Fair or poor health status	22.6	25.1	21.4
No health insurance coverage	8.1	16.5	3.9
No primary care provider	11.0	22.2	5.4
≥12 months since routine checkup or never had	19.5	33.8	12.4
Could not see a doctor because of cost	10.6	16.4	7.8
No physical activity	27.0	31.3	24.8
Binge drinking	10.9	12.6	10.1
Current smoker	16.1	22.7	12.8
BMI <30 kg/m^2^	38.2	38.0	38.4
Do not reside in metropolitan area	20.5	23.6	19.1
No. of chronic conditions			
0	31.1	38.9	27.2
1	29.3	28.4	29.7
2	19.2	16.1	20.8
3	10.2	8.1	11.2
≥4	10.2	8.4	11.1

a All values are weighted column percentages, unless otherwise indicated. Missing values: marital status (n = 3,704), education (n = 1,612), employment (n = 3,529), annual household income (n = 132,466), health status (n = 2,576), personal doctor (n = 2,795), last checkup (n = 8,170), medical cost (n = 2,278), exercise (n = 1,289), smoker (n = 5,265), binge drinking (n = 16,458), metropolitan status (n = 384,498).

b Hispanic adults who responded to the survey in Spanish were categorized as having limited English proficiency.

About 1 in 7 respondents had 4 or more chronic conditions (14.8% of non-Hispanic White respondents, 15.5% of non-Hispanic Black respondents, 14.0% of Hispanic respondents without LEP, and 14.8% of Hispanic respondents with LEP). Arthritis was the most common chronic condition among all racial and ethnic groups. The percentage of respondents with each chronic condition classified by the number of chronic conditions was similar across all racial and ethnic groups, except for diabetes; this percentage was higher among non-Hispanic Black respondents and both groups of Hispanic respondents than among non-Hispanic White respondents (Appendix Table 1 and 2). Adults with cancer, kidney disease, or arthritis tended to have higher rates of being up to date with colorectal cancer screening than adults with chronic obstructive pulmonary disease, depressive disorder, or cardiovascular disease (Appendix Table 3).

**Figure 1:**
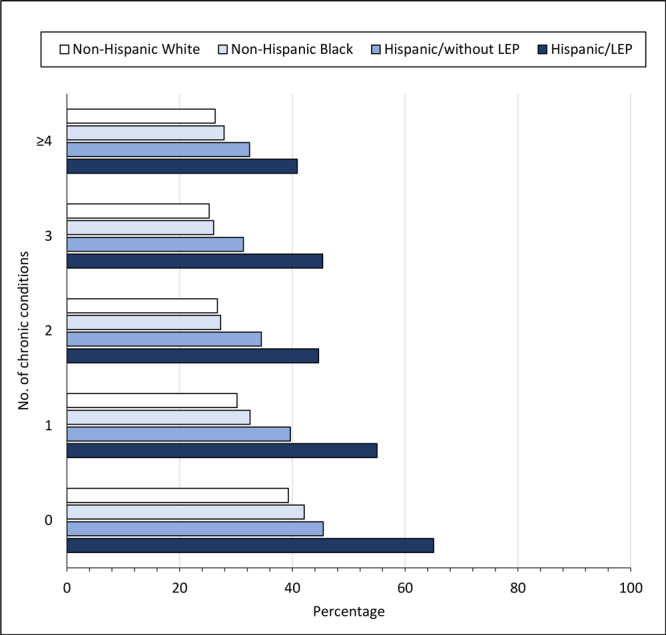
Percentage of adults who were not up to date with colorectal cancer screening by race and ethnicity and English-language proficiency, Behavioral Risk Factor Surveillance System, 2012–2020. Hispanic adults who responded to the survey in Spanish were categorized as having limited English proficiency (LEP). Non-Hispanic Black and non-Hispanic White groups were assumed to have English proficiency.

Regardless of race and ethnicity, the prevalence of being up to date with CRC screening increased as the number of chronic conditions increased. However, a higher percentage of Hispanic respondents with LEP were not up to date with CRC screening compared with all other racial and ethnic groups. Among Hispanic respondents with LEP, 65.1% with no chronic conditions and 40.9% with 4 or more conditions were not up to date with CRC screening (Figure 1).

**Table:** 

Race, ethnicity, language proficiency	No. of chronic conditions				
	0	1	2	3	≥4
Hispanic/limited English proficiency	65.1	55.0	44.6	45.4	40.9
Hispanic/no limited English proficiency	45.5	39.7	34.5	31.3	32.4
Non-Hispanic Black	42.1	32.5	27.3	26.0	27.9
Non-Hispanic White	39.3	30.2	26.7	25.2	26.3

**Figure 2:**
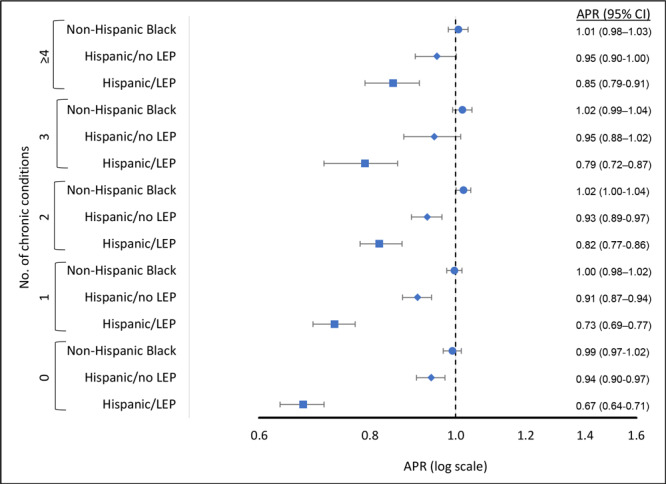
Use of colorectal cancer screening by race, ethnicity, English-language proficiency, and number of chronic conditions, Behavioral Risk Factor Surveillance System (BRFSS), 2012–2020. Adjusted prevalence ratios (APRs) were weighted according to BRFSS methodology. Estimates were obtained from a model that included an interaction term between the number of chronic conditions and race and ethnicity and were adjusted for age, sex, health insurance, and survey year. The reference group for all categories was non-Hispanic White. Hispanic people who responded to the survey in Spanish were categorized as having limited English proficiency (LEP). Error bars indicate 95% CIs.

After adjusting for potential confounders such as age, sex, health insurance, and survey year, among participants with no chronic conditions, Hispanic respondents with LEP had a 33% (APR = 0.67; 95% CI, 0.64–0.71) lower up-to-date CRC screening prevalence compared with non-Hispanic White respondents. Among respondents with 4 or more chronic conditions, Hispanic respondents with LEP had a 15% (APR = 0.85; 95% CI, 0.79–0.91) lower up-to-date CRC screening prevalence compared with non-Hispanic White respondents. Only Hispanic respondents without LEP with 0, 1, or 2 chronic conditions were less likely to be up to date with CRC screening than non-Hispanic White respondents, with prevalence ranging from 6% to 9%. We found no differences between non-Hispanic Black and non-Hispanic White respondents (Figure 2 and Appendix Table 4).

**Table:** 

No. of chronic conditions	Race and ethnicity	APR (95% CI)
≥4	Non-Hispanic Black	1.01 (0.98**–**1.03)
	Hispanic/no LEP	0.95 (0.90**–**1.00)
	Hispanic/LEP	0.85 (0.79**–**0.91)
3	Non**–**Hispanic Black	1.02 (0.99**–**1.04)
	Hispanic/no LEP	0.95 (0.88**–**1.02)
	Hispanic/LEP	0.79 (0.72**–**0.87)
2	Non-Hispanic Black	1.02 (1.00**–**1.04)
	Hispanic/no LEP	0.93 (0.89**–**0.97)
	Hispanic/LEP	0.82 (0.77**–**0.86)
1	Non-Hispanic Black	1.00 (0.98**–**1.02)
	Hispanic/no LEP	0.91 (0.87**–**0.94)
	Hispanic/LEP	0.73 (0.69**–**0.77)
0	Non-Hispanic Black	0.99 (0.97**–**1.02)
	Hispanic/no LEP	0.94 (0.90**–**0.97)
	Hispanic/LEP	0.67 (0.64**–**0.71)

## Discussion

We used BRFSS data from 2012 through 2020 to evaluate differences in CRC screening according to the number of chronic conditions among Hispanic respondents with and without LEP, non-Hispanic Black respondents, and non-Hispanic White respondents. Our results suggested that the likelihood of being up to date with CRC screening increased as the number of chronic conditions increased. However, we found significant differences in the association between being up to date with CRC screening and the number of chronic conditions across racial and ethnic groups. Hispanic respondents, particularly those with LEP, were less likely than non-Hispanic White respondents to be up to date with CRC screening. We observed no difference in adherence to CRC screening guidelines between non-Hispanic Black and non-Hispanic White respondents.

The relationship between use of CRC screening and number of chronic conditions is not well understood (32). Furthermore, to our knowledge, no previous studies have examined this association across racial and ethnic groups. Previous research showed inconsistent results, with higher rates of screening uptake linked to more frequent interactions with health care providers and lower rates of uptake attributed to the additional burden on patients of having to navigate the health care system with multiple conditions (16,17). We observed that Hispanic respondents, particularly those with LEP, were less likely than non-Hispanic White respondents to be up to date with CRC screening, even when we considered the number of chronic conditions. A previous study of the medical records of 3,433 adults aged 55 years or older conducted in 4 rural primary care clinics in Oregon found that adults with 3 chronic conditions were less likely than adults without any chronic conditions to be up to date with CRC screening (33). Another study, conducted in Oregon and California (36,208 participants; mean age, 59 years; 54% women), found that patients with any chronic disease were approximately 20% less likely to complete a fecal immunochemical test than patients without any chronic disease (34).

The disparity in CRC screening rates between Hispanic respondents, particularly those with LEP, and non-Hispanic White respondents was more pronounced among Hispanic people with none or just a few chronic conditions, which raises the question of why larger numbers of chronic conditions appear to reduce this disparity. A plausible explanation could be the influence of health care providers and their recommendations. It is well documented that health care providers play a pivotal role in advising patients on the importance of cancer screenings (35). Thus, people with chronic conditions (vs people with none) may have more frequent interactions with health care professionals who advise and promote CRC screenings as part of their comprehensive care plans, thereby reducing the disparity in screening rates (35).

Another important factor in CRC screening rates is age. The number of chronic conditions differed between adults aged 50 to 65 years and adults aged 65 years or older, thereby influencing CRC screening rates (14). In our study, we observed a larger number of chronic conditions among adults aged 65 years or older. To account for this, we adjusted for age in our final model. However, the dynamics of these age-related differences by race and ethnicity are not completely understood.

The difference in CRC screening rates among Hispanic people with and without LEP highlights the potential effect of English proficiency on health care disparities in this population. Other studies have observed disparities in the use of preventive care services among Hispanic people with LEP. For example, a study that used data from the Medical Expenditure Panel Survey found that Hispanic people with LEP faced challenges in education, employment, income, and health insurance and were less likely than Hispanic people without LEP to use health care services or have a usual source of care (36). The study also reported that Hispanic people with LEP (48%) were less likely than Hispanic people without LEP (68%) to receive CRC screening, but the differences were largely explained by age, education, marital status, health insurance coverage, time in the US, and survey year (36).

In contrast, our study found lower CRC screening rates among all Hispanic people compared with non-Hispanic White people, although we also considered the number of chronic conditions. Our findings were consistent with the findings of 2 other studies. First, an investigation of the relationship between limited language proficiency and CRC screening found that people with LEP (vs people without LEP) had lower rates of screening colonoscopies, had fewer polyps removed, and received fewer physician recommendations for colonoscopies. Spanish-speaking people were particularly at risk for lower screening rates (37). Second, a study using the 2019 National Health Interview Survey found that health insurance coverage contributed to, but did not completely explain, the observed inequality in CRC screening between non-Hispanic White people and Hispanic people. Although other sociodemographic factors may contribute to the disparity, unmeasured barriers may explain the differences (7).

### Strengths and limitations

Our study has several strengths, including the use of contemporary data from BRFSS, a comprehensive survey of health indicators that is generalizable to the US population, and the ability to examine multiple CRC screening testing modalities and investigate the role of chronic conditions and race and ethnicity in the use of screening tests while adjusting for potential confounders. However, the study also has limitations, and these limitations could potentially affect the interpretation of our results. One limitation is the reliance on self-reported information about use of CRC screening and chronic conditions, which may introduce recall bias. Additionally, the number and type of chronic conditions in this study was limited by the information available in the corresponding survey years. Furthermore, because of the small sample size of participants who answered the BRFSS survey in languages other than English, our ability to assess the effect of LEP on other racial and ethnic groups is limited. This limitation may have resulted in an incomplete understanding of how language barriers may influence CRC screening disparities among various racial and ethnic populations. Another limitation is the absence of certain important variables, such as geographic location, which can play a crucial role in health care access and disparities in use of health care services. The absence of these variables hindered our ability to provide a comprehensive analysis of the factors influencing CRC screening disparities. Finally, our findings on the relationship between chronic conditions and being up to date with any CRC screening may not necessarily reflect the findings for the relationship between chronic conditions and the various modalities of CRC screening, such as fecal immunochemical test, FOBT, sigmoidoscopy, or colonoscopy.

### Conclusions

Our findings have important implications for health care providers and policy makers aiming to improve screening rates and prevent CRC deaths (38,39). We identified access to health care as a crucial factor in screening rates: people who lacked health care coverage or a primary care provider (vs people who had both) or people who reported financial barriers to care (vs people who had no such barriers) were less likely to be up to date with CRC screening. Our study highlights the importance of considering chronic conditions when developing screening interventions and improving access to health care to reduce CRC-related health disparities, particularly among Hispanic people. Efforts to improve access to screening and address cultural and language barriers may also help reduce disparities in use of CRC screening and ultimately reduce CRC incidence and mortality. Future research is needed to explore the underlying factors contributing to these disparities and develop effective interventions to address them.

## Appendix

**Appendix Table 1:** Weighted Percentage of non-Hispanic White and non-Hispanic Black Adults With Any of 9 Chronic Conditions, Behavioral Risk Factor Surveillance System (BRFSS), 2012–2020^a^

Chronic condition	Non-Hispanic White, weighted %					Non-Hispanic Black, weighted %				
	Overall	1	2	3	≥4	Overall	1	2	3	≥4
Overall	—	42.3	28.1	14.8	14.8	—	41.1	28.2	15.3	15.5
Arthritis	60.3	42.5	65.8	78.05	84.1	62.6	43.7	68.1	80.7	85.9
Depressive disorder	28.7	11.6	30.2	45.0	59.3	24.5	7.4	22.8	40.1	58.8
Diabetes	22.3	9.8	21.6	34.9	47.1	38.1	22.5	40.5	53.3	60.4
Cardiovascular disease	19.2	6.5	16.9	30.5	48.7	22.4	7.1	20.1	36.7	53.3
Asthma	18.6	7.4	15.9	28.6	46.2	22.1	8.7	19.4	32.7	52.6
Skin cancer	17.7	10.6	19.3	24.8	28.2	1.1	0.3	0.8	1.4	3.2
Chronic obstructive pulmonary disease	15.6	3.1	10.9	26.0	51.1	14.2	2.2	9.7	21.9	48.2
Cancer	15.5	7.6	15.8	23.1	29.8	12.9	6.6	12.7	19.0	24.4
Kidney disease	5.6	1.0	3.6	9.1	19.5	8.2	1.5	5.9	14.3	24.8

Abbreviation: —, does not apply.

^a^ Values were weighted according to BRFSS methodology.

**Appendix Table 2:** Weighted Percentage of Hispanic Adults With Any of 9 Chronic Conditions, by English Language Proficiency, Behavioral Risk Factor Surveillance System (BRFSS), 2012–2020^a^

Chronic conditions	Not limited in English proficiency					Limited English proficiency				
	Overall	1	2	3	≥4	Overall	1	2	3	≥4
Overall	—	44.2	27.4	14.4	14.0	—	45.9	25.4	13.9	14.8
Arthritis	55.9	37.2	64.5	74.4	80.7	49.9	31.7	58.2	71.4	74.2
Depressive disorder	28.3	10.9	29.7	46.4	62.7	30.6	10.6	33.9	55.5	65.7
Diabetes	37.0	23.6	38.0	54.5	59.5	44.7	35.0	46.7	58.2	59.3
Cardiovascular disease	20.0	6.9	18.9	33.0	50.6	18.7	6.9	18.6	32.9	42.5
Asthma	21.5	9.8	19.5	36.7	47.0	18.5	6.7	14.4	33.1	49.1
Skin cancer	4.2	2.1	5.0	5.3	8.3	2.6	1.0	3.5	2.9	6.2
Chronic obstructive pulmonary disease	11.5	1.7	7.3	20.8	43.1	9.3	1.4	6.9	17.1	32.1
Cancer	11.6	6.5	12.7	14.3	22.7	9.2	4.5	9.8	12.2	19.9
Kidney disease	7.2	1.2	4.3	14.4	24.8	8.6	2.1	7.9	16.8	22.9

Abbreviation: —, does not apply.

^a^ Values were weighted according to BRFSS methodology. Hispanic adults who responded to the survey in Spanish were categorized as having limited English proficiency.

**Appendix Table 3:** Weighted Percentage of Adults With Any of 9 Chronic Conditions, by Use of Colorectal Cancer Screening, Behavioral Risk Factor Surveillance System (BRFSS), 2012–2020^a^

Chronic conditions	Any colorectal cancer screening, weighted %	
	Never or not up to date	Up to date
Chronic obstructive pulmonary disease	31.0	69.0
Depressive disorder	30.5	69.5
Cardiovascular disease	30.5	69.5
Asthma	30.2	69.8
Diabetes	30.0	70.0
Arthritis	27.3	72.7
Kidney disease	25.8	74.2
Skin cancer	21.0	79.0
Cancer	20.9	79.1

^a^ Values were weighted according to BRFSS methodology.

**Appendix Table 4:** Crude Prevalence Ratios for Use of Colorectal Cancer Screening by Race and Ethnicity and Number of Chronic Conditions, Behavioral Risk Factor Surveillance System (BRFSS), 2012–2020^a^

No. of chronic conditions	Race and ethnicity	Crude PR (95% CI)
≥4	Non-Hispanic Black	0.98 (0.95–1.00)
	Hispanic/no LEP	0.92 (0.87–0.97)
	Hispanic/LEP	0.80 (0.75–0.86)
3	Non-Hispanic Black	0.99 (0.96–1.02)
	Hispanic/no LEP	0.92 (0.86–0.99)
	Hispanic/LEP	0.73 (0.67–0.80)
2	Non-Hispanic Black	0.99 (0.97–1.01)
	Hispanic/no LEP	0.89 (0.86–0.93)
	Hispanic/LEP	0.76 (0.71–0.80)
1	Non-Hispanic Black	0.97 (0.95–0.99)
	Hispanic/no LEP	0.86 (0.83–0.90)
	Hispanic/LEP	0.64 (0.61–0.68)
0	Non-Hispanic Black	0.95 (0.93–0.98)
	Hispanic/no LEP	0.90 (0.86–0.93)
	Hispanic/LEP	0.58 (0.54–0.61)

Abbreviations: LEP, limited English proficiency; PR, prevalence ratio.

^a^ The reference group for all categories was non-Hispanic White. Hispanic people who responded to the survey in Spanish were categorized as having LEP.
